# Fatal acute Chagas disease by *Trypanosoma cruzi* DTU TcI, Ecuador

**DOI:** 10.1186/s12879-020-4851-0

**Published:** 2020-02-14

**Authors:** Manuel Calvopina, Gabriela Segovia, William Cevallos, Yosselin Vicuña, Jaime A. Costales, Angel Guevara

**Affiliations:** 1 OneHealth Research Group, Carrera de Medicina, Facultad de Ciencias de la Salud, Universidad de Las Américas (UDLA), Calle Jose Queri s/n entre Av. Granados y Av. Eloy Alfaro, PO BOX 17-17-9788, Quito, Ecuador; 2 Instituto de Biomedicina, Carrera de Medicina, Facultad de Ciencias Médicas, Universidad Central del Ecuador, Quito, Ecuador; 3 Centro de Investigación para la Salud en América Latina (CISeAL), Escuela de Ciencias Biológicas, Pontificia Universidad Católica del Ecuador, Quito, Ecuador

**Keywords:** *Trypanosoma cruzi*, TcI, DTU, Fatal acute Chagas disease, Ecuador

## Abstract

**Background:**

Chagas disease is caused by the haemoflagellate protozoan *Trypanosoma cruzi*. Currently, *T. cruzi* recognizes seven discrete typing units (DTUs): TcI to TcVI and Tcbat. The genetic diversity of *T. cruzi* is suspected to influence the clinical outcome. Acute clinical manifestations, which include myocarditis and meningoencephalitis, are sometimes fatal; occur most frequently in children and in immunocompromised individuals. Acute disease is often overlooked, leading to a poor prognosis.

**Case presentation:**

A 38-year-old man from a subtropical area of the Andes mountains of Ecuador was hospitalized after 3 weeks of evolution with high fever, chills, an enlarged liver, spleen, and lymph nodes, as well as facial edema. ECG changes were also observed. *T. cruzi* was identified in blood smears, culture and amplification of DNA by PCR. Tests for anti-*T. cruzi* IgG and IgM and HIV were negative. Molecular typing by restriction fragment length polymorphism (PCR-RFLP) determined the parasite to DTU TcI. In the absence of a timely anti-*T. cruzi* medication, the patient died.

**Conclusions:**

This is a case of severe pathogenicity and the virulence of a DTU TcI strain in an adult patient. The severe acute Chagas disease was probably overlooked due to limited awareness and its low incidence. Our findings suggest that *T. cruzi* DTU TcI strains circulating in Ecuador are capable of causing fatal acute disease. Early diagnosis and prompt treatment is of paramount importance to avoid fatalities in acute infections.

## Background

Data from the World Health Organization indicate that 6–7 million individuals are infected by *Trypanosoma cruzi,* from the south of the United States to the north of Argentina and Chile, with approximately 60–70 million people at risk of infection; Chagas disease is considered a neglected tropical disease [1].

Infected individuals may present with clinical manifestations of different levels of severity. The genetic diversity of *T. cruzi* is suspected to influence the clinical outcome although no definitive associations have been unequivocally identified [2, 3]. Seven genetic lineages or discrete typing units (DTUs) are currently recognized, named TcI through TcVI and Tcbat [3]. All lineages are present throughout the Americas, although TcI predominates in northern region of South America, and occurs in both domestic and sylvatic cycles of the parasite [2]. In addition, TcI infections are frequently reported in the northern section of the Amazon, where patients can display severe acute clinical manifestations, including cardiomyopathy and death [2]. TcI has also been isolated from chronic chagasic cardiomyopathy and reactivation disease [4]. TcII, TcV, and TcVI are associated with chronic cases presenting megaesophagus and megacolon in the southern cone countries [5, 6].

The acute phase of Chagas disease is usually asymptomatic; however, when symptoms do occur, they may include high fever, malaise, enlargement of the liver, spleen, and lymph nodes; as well as subcutaneous edema (localized or generalized) [7]. ECG changes are also common [2]. Death can occur in the acute phase (< 5–10% of symptomatic cases) due to severe myocarditis, meningoencephalitis, or both [7–9] and in some cases due to acute kidney failure (AKF) [10]. In addition, fatal cases with disseminated and diffuse foci have been observed [5]. Severe acute Chagas disease and related deaths occur most frequently in children, the elderly and immunocompromised individuals or in those who are receiving immunosuppressive drugs or organ transplants [4, 6, 7].

Chagas disease is endemic in Ecuador, country located in the northwest region of South America. TcI is clearly the predominant DTU, and has been the only DTU isolated from triatomines, rodents and opossums in the central Pacific coast and in southern Ecuador [4, 11]. However, a couple of reports exist in the literature suggesting the presence of genetic lineages other than TcI in Ecuador [12, 13]. *T. cruzi* infection is considered a public health problem in Ecuador, because it is endemic in the Amazon, the Pacific coast and in some subtropical areas of Andes mountains [7, 14]. The Pan-American Health Organization (PAHO) and the Ecuadorian Ministry of Public Health (MPH) estimate the general prevalence of *T. cruzi* infection to be 1.38% of the general Ecuadorian population with annual mortality of 7.7 per 1000 seropositive, meaning that there are 1300 deaths annually due to Chagas [http://chagas.zoonosis.gub.uy/Documentos/Ecuador/ Control_dela_Enfermedad_de_Chagas_en_Ecuador_OPS_Chagas.pdf. CP 17-1106292]. However, information is scarce regarding the genetic diversity of human isolates. Herein, we report a case of Chagas disease in a 38-year-old man who died in the acute phase due to myocarditis and renal failure. The patient was infected in the western foothills of the Andes close to the central Pacific coastal region, and the parasite isolated from his blood was determined to belong to *T. cruzi* DTU TcI.

## Case presentation

A farmer of 38-year-old man was admitted to a hospital with fever (39 °C), chills, malaise, anorexia, generalized pallor, hepatosplenomegaly, lymphadenopathy, weight loss, facial edema, and an ulcerous skin lesion in his left leg. The patient was born and always lived in a locality of La Maná-Cotopaxi province; a subtropical area located in the western foothills of the Andes, close to the central Pacific coastal region, some 130 km from the capital Quito. The patient reported neither receiving blood transfusions nor travelling within or outside Ecuador.

Since December 2014, he had a 22-day history of high fever, chills and malaise being treated with antibiotics and antipyretics in a public health center. Five days prior to hospital admission, he was diagnosed with *T. cruzi* infection via microscopic observation in peripheral blood. Due to unavailability of anti-*T. cruzi* drugs, he was transferred to a hospital. The patient reported of having an insect bite in his left leg approximately 10 days prior to the development of the fever. The bite became a pruritic indurated papule, which subsequently ulcerated and did not heal even with the use of an antibiotic cream. The patient was hospitalized with a diagnosis of acute Chagas disease, in order to receive treatment with benznidazole, the drug recommended by the Ecuadorian MPH [14].

At the beginning of hospitalization, microscopic examination of thick and thin smears of peripheral blood confirmed the presence of *T. cruzi,* and was negative for malaria parasites. *T. cruzi* was cultured in LIT medium. The parasites were spotted on FTA Classic Card (Whatman, Newton Center, MA). Blood tests showed a white blood cell count of 6.8 × 10^9^/L, with 44.8% neutrophils, 45.9% lymphocytes, 7.3% monocytes, 1.2% eosinophils and 0.8% basophils. The erythrocyte sedimentation rate was 40 mm/hour, with a hemoglobin of 8.4 g/dL, a hematocrit of 25.4%, and a platelet count of 152,000/μL. VDRL, HBsAg, HBcAg, HCV, ELISA and Western blot for HIV, and the test for febrile agglutinations (*Brucella* spp., typhoid and paratyphoid fevers), were all negative. Serum glucose, urea and creatinine were 77, 173 and 7.7 mg/dL, respectively. Serological tests for anti-*T. cruzi* IgG and IgM (Chagatest ELISA recombinant, Version 3.0. Wiener-Argentina) were negative. Blood, urine and skin ulcer cultures for bacteria were negative. Paracetamol (1 g) was administered every 8 h as an anti-febrile agent.

The EKG showed left anterior fascicular hemi block, QRS 0.10mms, marked deviation of the axis to the left (positive QRS in I and negative in AVF), small R waves and large S waves in III and AVF. The chest X-ray demonstrated an increased heart area. An abdominal CT scan showed bilateral pleural effusion, liver and spleen enlargement, and liquid in the pelvic cavity.

During the 11-day hospitalization period, the patient’s temperature fluctuated from normal to 38 °C. Hydration and electrolytes were normal. Creatinine levels initially rose to 10.4 and later to 13.1 mg/dL, requiring hemodialysis. The patient received six blood transfusions in total, elevating the hemoglobin to 11.8 g/dL. The patient died of respiratory distress due to acute failure of heart and kidneys. He never did receive benznidazole because of hospital shortage. Parents did not consent to an autopsy.

**Fig. 1:**
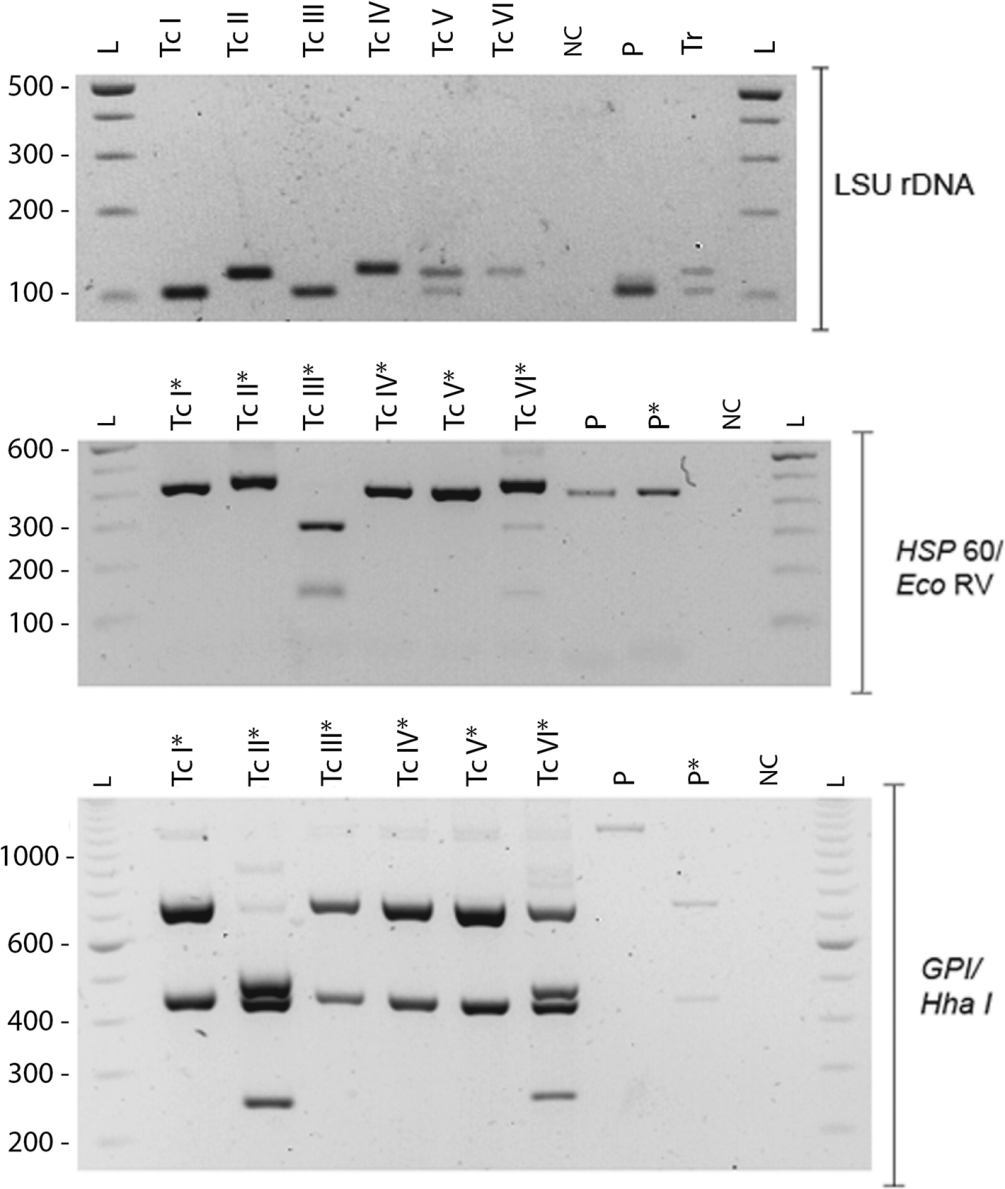
*T. cruzi* genotyping by PCR-RFLP. As indicated by the brackets on the right side, specific fragments from the LSUr DNA, *HSP*60 and *GPI* genes were amplified by PCR. *GPI* and *HSP*60 amplification products were digested with *Hha*I and *Eco*RV restriction enzimes, respectively. **L**: DNA molecular weight ladder, with the corresponding molecular weights in base-pairs indicated on the left of the gels. **TcI-TcVI**: DTU controls. **N:** Negative (no template) control. **P**: DNA isolated from the patient’s blood. **Tr**: *T. rangeli* DNA. Lanes containing restriction products are labeled with an asterisk (*). Only restriction products are shown for controls

**Fig. 2:**
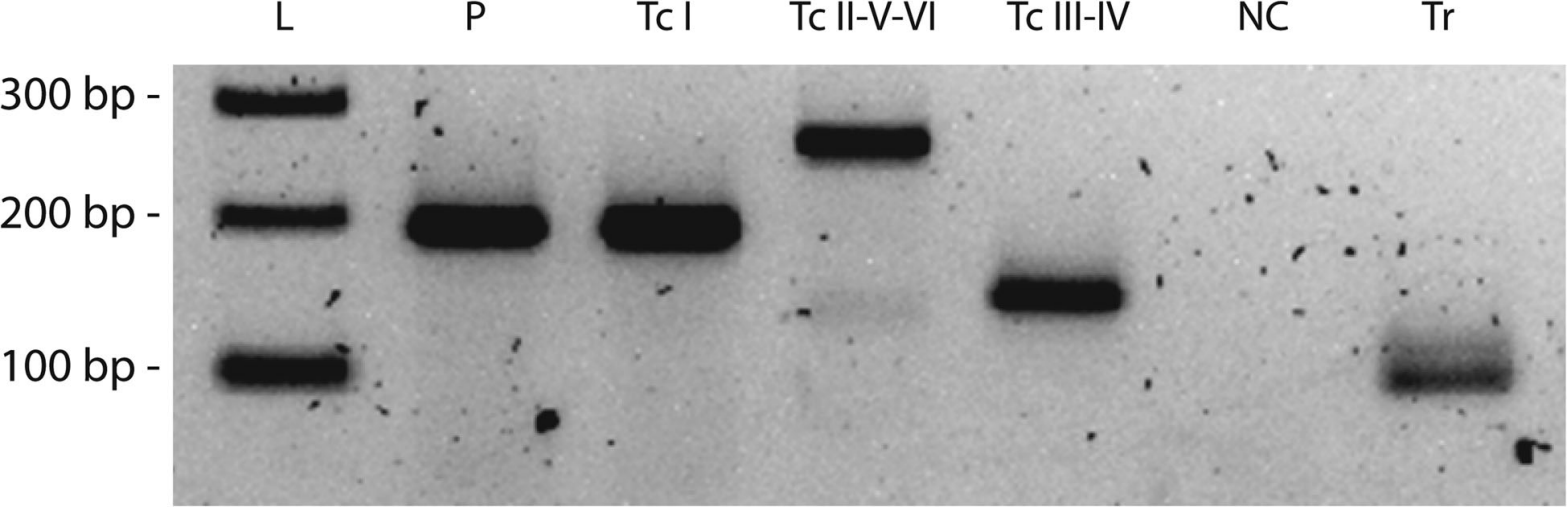
*T. cruzi* genotyping by mini-exon multiplex PCR. Parasite genotyping was performed using the multiplex PCR assay targeting the mini-exon gene, which differentiates TcI from the other *T. cruzi* DTUs. **L**: DNA molecular weight ladder, with the corresponding molecular weights in base-pairs indicated on the left of the gel. **P**: DNA isolated from parasites cultured from the patient’s blood. **TcI.** DTU TcI control DNA (Cutia Cl1 strain), yielding a 200 bp band. **TcII-V-VI.** Control DNA from DTU TcII DNA (Tu18Cl93 strain), yielding a 250 bp band, corresponding to DTUs TcII, V or VI. **TcIII-IV**. DTU TcIV (CAN III Cl1 strain), yielding a 150 bp band, corresponding to DTUs III or IV. **NC.** Negative (no template) control. **Tr**. *T. rangeli* control DNA, yielding a 100 bp band

*T. cruzi* DNA was extracted from the FTA card. Genotyping was performed by PCR-RFLP, following the methodology developed by Lewis et al. (2009) [15]. Briefly, fragments of the D7 divergent domain of the 24Sα rRNA locus (LSU rDNA), glucose-6-phosphate isomerase (*GPI*) and heat shock protein 60 (*HSP60*) genes were amplified with specific primers; the size of amplicons and restriction fragments after digestion with restriction enzymes (*Eco* RV for HSP60 and *Hha* I for GPI) were compared to those of reference *T. cruzi* strains (Fig. 1). Additionally, a multiplex PCR assay targeting the mini-exon gene [16] was also performed (Fig. 2). In both cases, the *T. cruzi* isolate from the patient matched the pattern of DTU TcI. Written consent to publish the case was provided by the patient’s wife.

## Discussion and conclusions

This is the first time in Ecuador to genotype *T. cruzi* TcI (DTU) from a human source, although the patient did not survive in the acute phase of the Chagas disease. To date, TcI is the predominant DTU in Ecuador, as evidenced in studies but involving reservoirs and vectors from different geographic regions [4, 11]. It is in concordance with previous reports from the northern region of South America that showed DTU TcI being predominant in the neighboring countries of Colombia and Venezuela [17, 18]. It has been hypothesized that TcI can escape the host’s acute immune response, remain in the peripheral blood mononuclear cells and then parasitize organs faster [5, 19]. The present case suggests that the TcI strain circulating in the country are capable of causing fatal acute disease. Hence, further research is needed to identifying the genetic lineages of *T. cruzi* DTU in different clinical presentations and the severity of the Chagas disease.

Acute Chagas cases resulting in death have been documented, mostly from oral contamination and found most frequently in the Amazon region [20]. Most of these cases were due to TcI, with rare cases due to TcIII and TcIV [21–23], TcI in Venezuela and French Guiana [24, 25] and TcII in southern Brazil [26]. However, in this case, the infection apparently occurred via the vectorial route, as suggested by the presence of a chagoma, i.e. the pruritic papular lesion that evolved into an ulcer in the left leg, starting 10 days before the appearance of fever and malaise. The presence of *T. cruzi* vectors has been reported in Cotopaxi province, specifically *Triatoma carrioni* and *T. dispar* [27].

Symptoms frequently reported in acute fatal cases of Chagas disease are hepatomegaly (100%), myocarditis (75%), pericardial effusion (50%), cardiomegaly (25%) or acute kidney failure (AKF) [10, 17, 20]. In the present case, the patient was clearly in the acute phase, because of the duration of the symptoms and the absence of anti-*T. cruzi* IgM and IgG antibodies. The absence of antibodies would be explained because 1) In order to develop detectable antibodies, it generally takes at least 3 to 4 weeks [28]. 2) In acute cases reported in Venezuela, specific IgM antibodies were demonstrated only in 87.3% of cases, and the transmission was oral, that is considered more severe, because of the rapid entry of parasites into the blood stream [24]. Hence, prompt development of antibodies can occur when compared to a natural infection caused by a triatomine bite, as probably occurred in our case. 3) Another explanation could be because of the different antigens used in the ELISA techniques performed, in-house with the delipidised antigen specific for *T. cruzi* epimastigotes [24] would detect prompt antibodies rather than we used the commercially ELISA method Chagatest ELISA recombinant, Version 3.0. Wiener-Rosario, Argentina, based on six recombinant proteins. Severe symptomatology is known to occur in immunocompromised patients [7]. However, our patient had no history of taking immunosuppressive drugs and was HIV-negative. The symptoms present in our patient including edema, EKG alterations, cardiomegaly, bilateral pleural effusion and the elevation of creatinine and blood urea nitrogen, strongly suggested acute myocarditis and AKF. The latter condition is usually marked by a rise in serum creatinine concentration or by azotemia.

High mortality in acute cases of Chagas disease has been linked to lack of prompt diagnosis and treatment. Both may have contributed to worsening of the patient’s condition and his subsequent death. We strongly advise health-care providers, lab technicians, physicians, as well as decision-makers in the central health entities to increase awareness about Chagas disease, and to improve the availability and distribution of anti-*T. cruzi* medication in the country. This case exemplifies the challenges faced by the local healthcare system in this regard, and constitutes an urgent call for action in order to ensure early diagnosis and prompt treatment is available to all chagasic patients in Ecuador.

## Data Availability

All relevant data and materials are included in the manuscript.

## Abbreviations

AKF: Acute kidney failure
DTUs: Discrete typing units
EKG: Electrocardiogram
ELISA: Enzyme-linked immunosorbent assay
HBcAg: Anti-core hepatitis
HBsAg: Hepatitis B surface antigen
HCV: Hepatitis C Virus
HIV: Human Immunodeficiency Virus
MPH: Ministry of Public Health
PAHO: Pan American Health Organization
PCR-RFLP: Polymerase Chain Reaction-Restriction fragment length polymorphism
VDRL: Venereal Disease Research Laboratory test
WHO: World Health Organization
